# Unveiling the Hidden Patterns: Multimodality Cardiac Imaging in Takotsubo Cardiomyopathy Diagnosis

**DOI:** 10.14797/mdcvj.1288

**Published:** 2023-11-16

**Authors:** Moath Said Alfawara, Mahmoud Al Rifai, Malek Nayfeh, Mohammed A. R. Chamsi-Pasha, Mouaz H. Al-Mallah

**Affiliations:** 1 Houston Methodist DeBakey Heart & Vascular Center, Houston Methodist Hospital, Houston, Texas, US

**Keywords:** Takotsubo cardiomyopathy, cardiac echocardiography, cardiac computed tomography, cardiac positron emission tomography, cardiac magnetic resonance imaging

## Abstract

Takotsubo cardiomyopathy, also known as stress cardiomyopathy, is a reversible form of cardiomyopathy characterized by reduced ejection fraction with regional wall motion abnormalities, elevated cardiac enzyme levels, and signs of ischemia on electrocardiogram despite the absence of obstructive epicardial coronary artery disease. It is often preceded by intense emotional or physical illness stressors. This case describes a 65-year-old female patient who likely developed takotsubo cardiomyopathy precipitated by the stress of diverticulitis.

## Introduction

Takotsubo cardiomyopathy, also known as stress cardiomyopathy, is a reversible form of cardiomyopathy characterized by reduced ejection fraction with regional wall motion abnormalities (RWMA), elevated cardiac enzyme levels, and signs of ischemia on electrocardiogram despite the absence of obstructive epicardial coronary artery disease.^1^ It is often preceded by intense emotional or physical illness stressors.^2^

## Case

**Figure 1:**
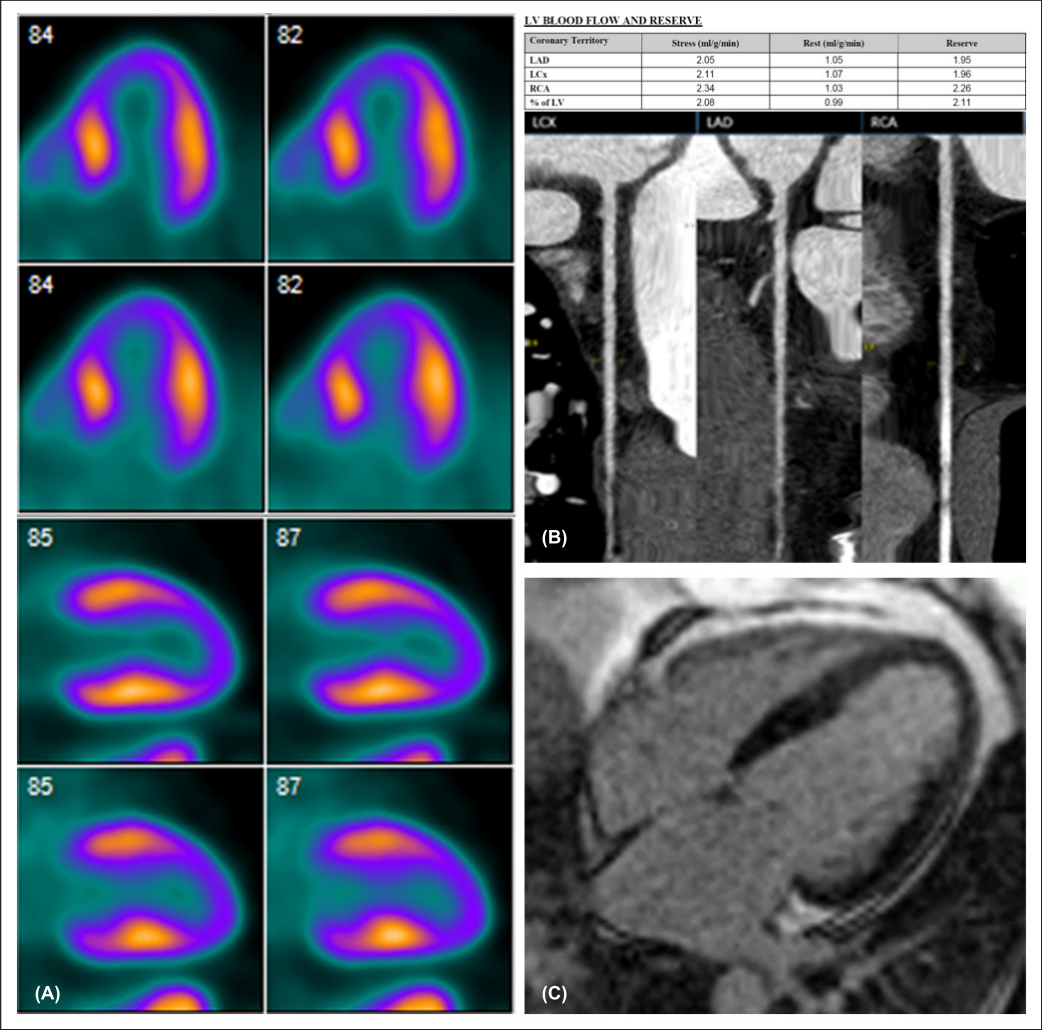
**(A)** Positron emission tomography cardiac images horizontal and vertical long axis shows a medium-sized severe fixed defect in the distal anterior, lateral, inferior, and septal segments and apex. **(B)** Cardiac computed tomography angiography 3-dimensional reconstruction shows coronary arteries without stenosis. Cardiac positron emission tomography myocardial blood flow/myocardial flow reserve (MFR) for the coronaries shows normal MFR. **(C)** Late gadolinium enhancement cardiac magnetic resonance imaging shows no evidence of myocardial infarction or scar.

A 65-year-old female patient, known to have bipolar disorder, hypertension, and diverticulosis was admitted to the hospital with symptoms of nausea, vomiting, and diarrhea suggestive of diverticulitis. Abdominal computed tomography (CT) confirmed the diagnosis, and antibiotics were administered, leading to partial resolution of her gastrointestinal symptoms. On the third day of admission, she started to complain of shortness of breath. Physical examination revealed diffuse crackles, and a chest x-ray demonstrated the presence of pulmonary edema. A transthoracic echocardiogram (Video 1) showed new apical and septal RWMA with a reduced ejection fraction (EF 55%).Hybrid cardiac positron emission tomography (PET)/CT was ordered to rule out ischemia (Figure 1A), and it revealed a medium-sized 22% fixed perfusion defect in the apex and apical segments. The coronary artery calcium score was zero. Gated images revealed hypokinesis of the apex and apical segment. Both regional and global myocardial blood flow was preserved as well as hyperemic myocardial blood flow, and coronary CT angiography (CCTA) confirmed the absence of obstructive coronary artery disease (Figure 1B). Cardiac magnetic resonance (CMR) imaging was subsequently performed. CMR cine steady-state free precession sequence (Video 2) and late gadolinium enhancement study (Figure 1C) showed normal left ventricle size and systolic function (EF 64%) but with RWMA in the apical and apical/septal segments, indicative of recovering cardiomyopathy given the absence of myocardial scarring and presence of edema. Our patient likely developed takotsubo cardiomyopathy precipitated by the stress of diverticulitis.

**Video 1:** Echocardiography left ventricle (LV) 4-chamber view shows mildly enlarged LV size with apical and septal regional wall motion abnormalities; see also at https://youtu.be/y6DVTtyomQc.

**Video 2:** Steady-state free precession cine cardiac magnetic resonance imaging shows normal left ventricle size and systolic function with mild regional wall motion abnormalities in the apical/apical septal segments; https://youtu.be/gD6p9J_3euU.

Some studies have shown that takotsubo cardiomyopathy is more common in patients with bipolar disorder.^3^ Stress cardiomyopathy can result in myocardial edema, causing transient left ventricular diastolic dysfunction and increased filling pressures with subsequent higher coronary perfusion pressures.^4^ This may explain the fixed relative hypoperfusion defects on PET/CT static images. The absence of calcified coronary artery plaque and normal myocardial blood flow on PET/CT dynamic images raised the suspicion of a nonischemic etiology for the observed perfusion defects, which was further confirmed on CCTA and CMR. The recovery period of takotsubo cardiomyopathy is typically a few days to weeks, but there are long-term clinical consequences.^5^
